# Dependence of InGaN Quantum Well Thickness on the Nature of Optical Transitions in LEDs

**DOI:** 10.3390/ma15010237

**Published:** 2021-12-29

**Authors:** Mateusz Hajdel, Mikolaj Chlipała, Marcin Siekacz, Henryk Turski, Paweł Wolny, Krzesimir Nowakowski-Szkudlarek, Anna Feduniewicz-Żmuda, Czeslaw Skierbiszewski, Grzegorz Muziol

**Affiliations:** Institute of High Pressure Physics, Polish Academy of Sciences, Sokolowska 29/37, 01-142 Warsaw, Poland; mik@unipress.waw.pl (M.C.); msiekacz@unipress.waw.pl (M.S.); henryk@unipress.waw.pl (H.T.); wolny@unipress.waw.pl (P.W.); krzesimir.szkudlarek@unipress.waw.pl (K.N.-S.); ania_f@unipress.waw.pl (A.F.-Ż.); czeslaw@mail.unipress.waw.pl (C.S.); gmuziol@unipress.waw.pl (G.M.)

**Keywords:** InGaN, nitrides, light-emitting diode, quantum well, molecular beam epitaxy

## Abstract

The design of the active region is one of the most crucial problems to address in light emitting devices (LEDs) based on III-nitride, due to the spatial separation of carriers by the built-in polarization. Here, we studied radiative transitions in InGaN-based LEDs with various quantum well (QW) thicknesses—2.6, 6.5, 7.8, 12, and 15 nm. In the case of the thinnest QW, we observed a typical effect of screening of the built-in field manifested with a blue shift of the electroluminescence spectrum at high current densities, whereas the LEDs with 6.5 and 7.8 nm QWs exhibited extremely high blue shift at low current densities accompanied by complex spectrum with multiple optical transitions. On the other hand, LEDs with the thickest QWs showed a stable, single-peak emission throughout the whole current density range. In order to obtain insight into the physical mechanisms behind this complex behavior, we performed self-consistent Schrodinger–Poisson simulations. We show that variation in the emission spectra between the samples is related to changes in the carrier density and differences in the magnitude of screening of the built-in field inside QWs. Moreover, we show that the excited states play a major role in carrier recombination for all QWs, apart from the thinnest one.

## 1. Introduction

Since the early 1990s, when I. Akasaki, H. Amano and S. Nakamura achieved an efficient GaN-based light emitting diode (LED), the III-nitride optoelectronic conquered the light emission devices global market [1]. The use of InGaN active layer enables efficient light emission from the blue to green spectral range. The efficiency above 100 lm/W and high reliability allowed GaN-based LEDs to replace other general lighting devices, such as incandescent lamps [2,3]. Recent advancements have pushed the external quantum efficiency of III-N LEDs close to unity [4,5,6].

The III-nitride semiconductor family has extremely large spontaneous and piezoelectric polarization parameters [7]. In many cases, the polarization fields are used to eliminate critical problems or enhance the performance of the device. The two-dimensional electron gas AlGaN/GaN field effect transistor with high mobility of carriers was created, thanks to the presence of built-in polarization fields [8,9]. In III-nitride tunnel junctions, the piezo-fields can be used to increase the tunneling current [10,11,12]. Great limitation of the electron overflow over the quantum wells can be achieved by changing the alignment of the polarization fields, thanks to the tunnel junction placed on bottom of the structure [13,14]. The concept of doping via polarization fields was shown in various III-N devices [15,16,17]. However, in the case of standard III-N optoelectronic devices, the effect of the built-in polarization is unfavorable. Inside InGaN QWs, the built-in polarization is responsible for the spatial separation of electrons and holes wavefunctions and the quantum-confined Stark effect (QCSE) [18]. During operation, carriers start to fill the active region and screen the internal electric field, which causes a shift of emission spectra. Nonetheless, the field is not fully screened, even up to the high current densities of j = 10 kA cm^−2^ [19]. It was predicted theoretically that increase in the QW width causes significant separation of carriers and leads to extremely small oscillator strength [20,21]. Additionally, it was shown experimentally that with the increasing thickness for QW, the photoluminescence intensity drops [22,23]. This originally led to the usage of thin QW as an active region in nitride LEDs [20,23]. The problem of low wavefunction overlap was partially mitigated by using staggered QWs [24,25]. On the other hand, there are a few literature reports showing that electrically driven devices with wide QW can have higher efficiency than the thin QWs [26,27,28]. In particular, very recently, the room-temperature operation of the deep UV laser diode was reported, operating on a single 9 nm wide QW [29]. We showed that the reason behind the high efficiency of the wide QWs lays in the screening of the built-in electric field by carriers occupying the ground states and radiative transition through exited states with high wavefunction overlap [30]. Additionally, we reported on the utilization of wide InGaN QWs for laser diodes [31]. Brecha et al. showed that the piezoelectric polarization is present without excitation, even in QWs as thick as 25 nm [32]. On the other hand, Pieniak et al. showed that the polarization field in such wide QW is fully screened under excitation [33].

In this paper, we study the mechanism governing light emission from InGaN QWs of various widths. We present evidence for emission from excited states in the case of LEDs with sufficiently wide QWs. We investigated the radiative transitions in LEDs with a single QW varying its thickness from 2.6 nm to 15 nm. We show how the transition path changes from ground states into excited states as the QW width is increased. Interestingly, in the intermediate QW width regime, an interplay between recombination through ground and excited states can be observed, and mixed transition is identified. In order to show the evolution from ground state emission through mixed into excited state emission, an extremely broad current density range was used, spanning from 0.1 A cm^−2^ up to 1000 A cm^−2^. Additionally, we performed LED operation simulations to give a comprehensive understanding of the role of the ground and excited states.

## 2. Samples and Methods

**Figure 1:**
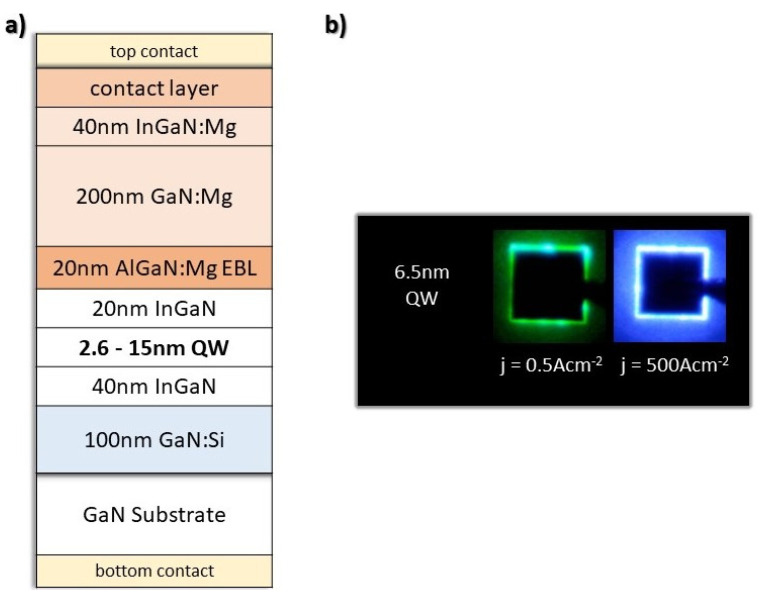
(**a**) Schematic structure of grown and simulated LEDs. (**b**) Example photographs of measured devices operating on intermediate single QW in low and high current density.

The InGaN LEDs were grown by plasma assisted molecular beam epitaxy (PAMBE). The growth of LEDs presented in this paper was conducted either in a VG V90 or a Veeco Gen20A rectors. Both of the reactors were equipped with two Veeco RF plasma sources in order to change the growth rate quickly. The growth of GaN layers was conducted at 730 °C in gallium-rich conditions with a growth rate of 0.36 µm h^−1^, while InGaN layers were grown in indium-rich conditions at 650 °C with a higher growth rate of 1 µm h^−1^. The higher growth rate in the case of InGaN was used to ensure a better optical quality of the QWs [34]. Further details of the PAMBE technology can be found in Ref. [35], while the InGaN growth model can be found in Ref. [36]. The structures were fabricated on freestanding GaN substrates with the threading dislocation density in the order of 10^7^ cm^−2^. All structures start with a 100 nm GaN doped with Si. Next, an undoped 40 nm In_0.02_Ga_0.98_N layer was grown followed by an In_0.17_Ga_0.83_N single QW with thickness equal to 2.6, 6.5, 7.8, 12 or 15 nm. This was followed by 20 nm undoped In_0.02_Ga_0.98_N and a highly doped 20 nm Al_0.13_Ga_0.87_N:Mg electron blocking layer (EBL). Next, the 200 nm p-type GaN:Mg and 40 nm In_0.02_Ga_0.98_N:Mg were grown. Lastly, a highly doped 5 nm In_0.14_Ga_0.86_N:Mg was grown to act as a contact layer. The doping concentrations were 2 × 10^18^, 3 × 10^19^, 1 × 10^18^, 2 × 10^19^, and 1 × 10^20^ cm^−3^ for the GaN:Si, EBL, GaN:Mg, In_0.02_Ga_0.98_N:Mg and In_0.14_Ga_0.86_N:Mg contact layers, respectively. A schematic of the grown structures is shown in Figure 1.

The samples were processed into 150 × 150 μm^2^ LED devices with 470 nm mesa height etched by reactive ion etching. A standard Ti/Al/Ni/Au 300/600/400/750 Å thick, metal contact was deposited on the backside of the substrate. On the top side, a Ni/Au 25/75 Å semi-transparent metallization was deposited. Next, it was covered with Ni/Au metallization with thickness 250/750 Å, leaving a 10 um margin to the edge of the etched mesa. This ensured even current spreading and a sufficient amount of light to be emitted from the device. The electroluminescence (EL) spectra were measured in DC operation in a wide drive current density range from 0.1 A cm^−2^ to 1000 A cm^−2^. The higher values of current are overheating the devices, which influences the device operation. The Drift–Diffusion Poisson–Schrodinger Solver Version created by Yuh-Renn Wu [37,38,39] was used to calculate band structures, compute the wavefunction overlap of the ground and excited states and simulate the theoretical spectra for the studied LEDs.

## 3. Results and Discussion

### 3.1. Experiment

**Figure 2:**
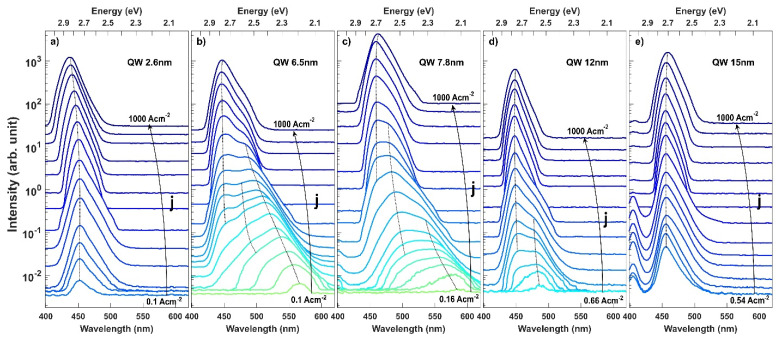
Selected measured electroluminescence spectra of InGaN LEDs utilizing QW with thickness ranging from (**a**) 2.6 nm, (**b**) 6.5 nm, (**c**) 7.8 nm, (**d**) 12 nm and (**e**) 15 nm for increasing drive current. The dotted lines indicate the peak emission wavelength from different states and are assigned arbitrarily.

**Figure 3:**
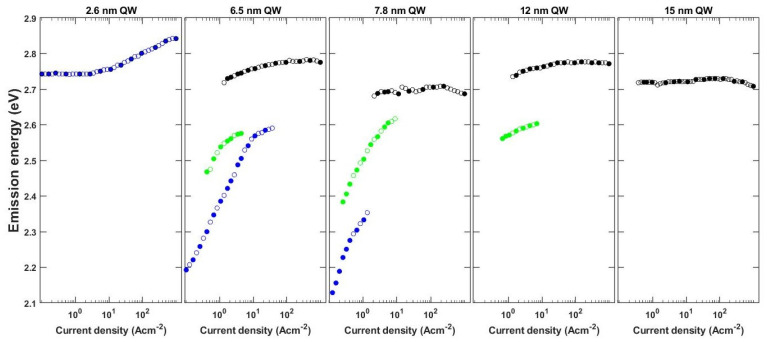
Dependence of the peak emission wavelength on current density for devices with various thicknesses of QW in the whole measured range of drive current.

We performed the experimental study of the EL spectra of the InGaN based LEDs in order to understand the recombination mechanism. The collected spectra are presented in Figure 2. In the case of LEDs with 6.5, 7.8 and 12 nm, we observed multiple peaks, which strongly shift and change their relative intensity with the supplied current. In order to better present this complex behavior, we extracted the dependence of the position of each peak on current density and show it in Figure 3. Based on the differences in the behavior we can group the LEDs into three categories: (i) thin QW–LED with 2.6 nm, (ii) intermediate QW–LED with 6.5 and 7.8 nm, and (iii) wide QW–LEDs with 12 and 15 nm.

In the case of the LED with the thin QW, we observed a single peak emission at λ = 452 nm, which starts to shift to shorter wavelengths above a current density of j = 4.4 A cm^−2^. This is a well-known behavior and is explained by screening of the built-in field by the supplied carriers, which decreases the QCSE. The magnitude of the blue shift at very high current densities is reduced by the heating of the device. The current required for the device operation in j = 1000 A cm^−2^ is equal to I = 225 mA and generates a non-negligible amount of Joule heat. The slight red shift of the emission is visible for all samples at high current density.

For LEDs with intermediate QW thicknesses a different behavior can be observed. The emission starts in the yellow color regime and is characterized by a very strong blue shift in the low current regime, which slows down as the current is increased. Additionally, multiple peaks can be resolved. In the case of LED with 6.5 nm QW we observe that the initial peak starts to emit at λ = 565 nm and strongly blue shifts. A second peak appears at λ = 495 nm and j = 0.4 A cm^−2^, followed by a third at λ = 457 nm and j = 1.3 A cm^−2^. The first peak shifts up to λ = 480 nm at a current density of j = 37 A cm^−2^, above which it stops to be easily distinguishable from the third peak. However, its remnants can be visible as a bump on the long wavelength side of the main peak. In the meantime, the second peak with a lower intensity on the short wavelength shoulder of the first peak stops to be distinguishable above j = 4.4 A cm^−2^ also due to the increased intensity of the third peak. Interestingly, the intensity of the third peak is strongly rising and conceals the positions of the first and second peaks. A qualitatively comparable evolution of spectra can be observed for LED with 7.8 nm QW. However, the relative intensities and currents at which the peaks appear and stop to be distinguishable differ. For example, the first starts to emit at λ = 582 nm, strongly blue shifts and vanishes at j = 1.3 A cm^−2^.

In case of the LEDs with wide QWs, yet another different behavior can be noticed. First of all, a higher current density is necessary in these LEDs to observe light emission. In our case, the LEDs started to emit detectable light at current densities of j = 0.16 A cm^−2^, j = 0.66 A cm^−2^ and j = 0.54 A cm^−2^ for LED with 7.8, 12 and 15 nm, respectively. In the case of LEDs with 2.6 nm and 6.5 nm QWs, the threshold to observe light is below j = 0.1 A cm^−2^. The LED with 12 nm QW starts to emit at λ = 485 nm. This peak slightly blue shifts and vanishes quickly in the high intensity of a second peak appearing at j = 1.3 A cm^−2^. The second peak shifts only slightly toward shorter wavelength as the current is increased. In the case of the LED with 15 nm QW, the spectra are dominated by a single peak, which does not shift throughout the whole current span. Only a small red shift is observed at currents higher than j = 300 A cm^−2^ due to heating.

The observed properties of the different QWs can be useful in various optoelectronic devices. For example, an LED with a current dependent emission wavelength can be produced with the use of intermediate QWs. The wide QW can be used as a wavelength-stable light source in high current bias application when the standard thin QW exhibits high peak shift of emission wavelength. In applications such as distributed feedback laser diodes, stable emission from device containing wide QW is very beneficial [40].

### 3.2. Simulation

**Figure 4:**
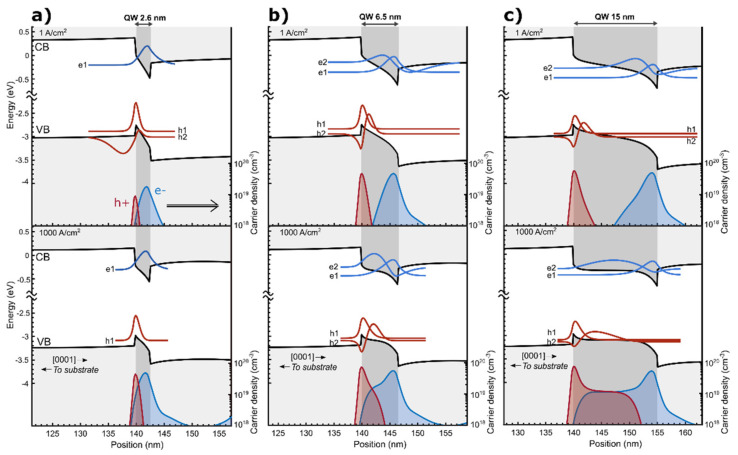
Calculated band structure of the LED with In_0.17_Ga_0.83_N QW with thickness (**a**) 2.6 nm (**b**) 6.5 nm and (**c**) 15 nm. The wave functions of the e1, e2, h1 and h2 states and density of holes and electrons are shown for low and high current density regimes.

In order to understand the recombination mechanism behind the complex behavior of the experimentally observed emission spectra, we performed the simulations of grown samples. Selected band profiles and wavefunctions together with the carrier distribution of the LEDs with thin, intermediate and wide QWs are shown in Figure 4a–c, respectively. Two cases are presented to discuss the LED operation in two extreme regimes: (i) low current density j = 1 A cm^−2^, and (ii) high current density j = 1000 A cm^−2^. In the low current density regime, the electrons and holes are spatially separated as a result of the built-in polarization fields. This separation is more evident for QWs with larger thickness. In the case of the intermediate and wide QWs, the built-in electric field is already partially screened by the large carrier density confined at the edges of the QWs.

An interesting change occurs in the behavior of the QW at high injection current. In the case of thin QW, only a slight reduction in the built-in electric field inside the QW can be observed, as a result of partial screening of the built-in field by the carriers located inside the QW. However, in the case of intermediate and wide QWs, qualitatively distinct changes occur. At high injection current, the electric field in the middle of these wells is almost entirely screened. The remnants of the built-in field can be still observed at the QW interfaces, due to the two-dimensional nature of polarization charges and three-dimensional distribution of mobile charge carriers. Lack of the electric field in the middle of the QW promotes homogenous carrier distribution. As can be seen in Figure 4c, at high injection, both the electrons and holes are present in the middle of the wide QW and can recombine with each other.

**Figure 5:**
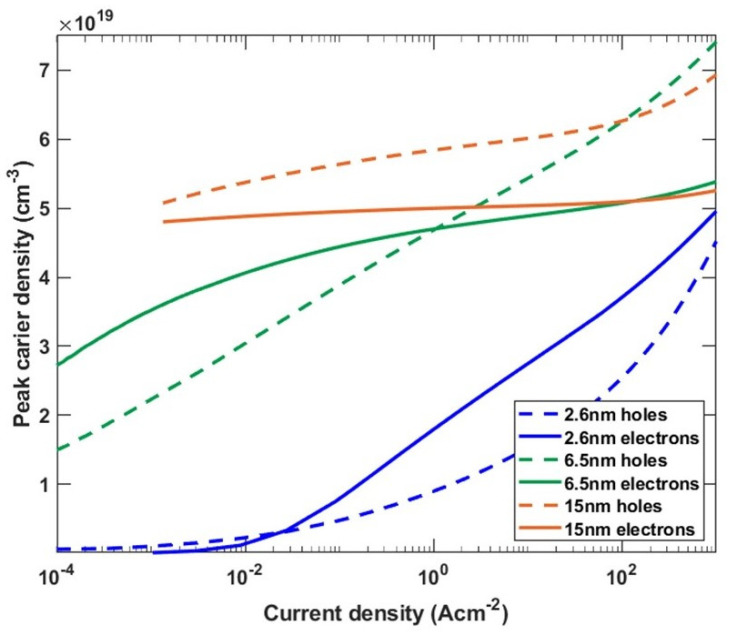
The peak density of holes and electrons in thin (blue), intermediate (green) and wide (red) QW.

The dependence of the carrier density located at the edges of the QW on current density, shown in Figure 5, is what clearly distinguishes the three regimes of the QW thickness. It is important to note that the values plotted in Figure 5 are the peak maxima of the separated electron and hole distributions. This dependence is presented to discuss the process of screening of the built-in field. In case of the thin QW, both the electron and hole densities are low in the low current regime and start to slowly rise as the current is increased. In the case of the intermediate QWs, the carrier density rises very rapidly already in the low current density regime. In contrast, in the wide QW, there is a large carrier density, even at very low currents, and there is only a slight increase at high currents. At the carrier densities observed in the intermediate and wide QWs, the excited states start to be occupied and play a significant role in carrier recombination.

The reason why the carrier densities in the three regimes of QW thickness have such a different dependence on current density lays in the difference in the recombination mechanism or rather an inefficient recombination path through the ground states in the case of intermediate QWs and almost a complete lack in the case of the wide QWs. It is worth mentioning that the steady state conditions require the recombination rate at a given current density to be the same for all of the LEDs. However, in order to maintain the same recombination rate, the occupation of the ground states in the case of the intermediate and wide QWs needs to be higher. In other words, the inefficient recombination path through the ground states promotes an increase in the carrier density. This situation changes with the screening of the piezoelectric field and appearance of the efficient transition through the excited states.

In order to study the influence of the QW width on carrier recombination, we calculated wavefunction overlaps between the two lowest electron and hole states. Importantly, due to breaking of the symmetry by the polarization charges at the interfaces, all four possible combinations of transitions from the electron (e1, e2) to the hole (h1, h2) states are permitted.

**Figure 6:**
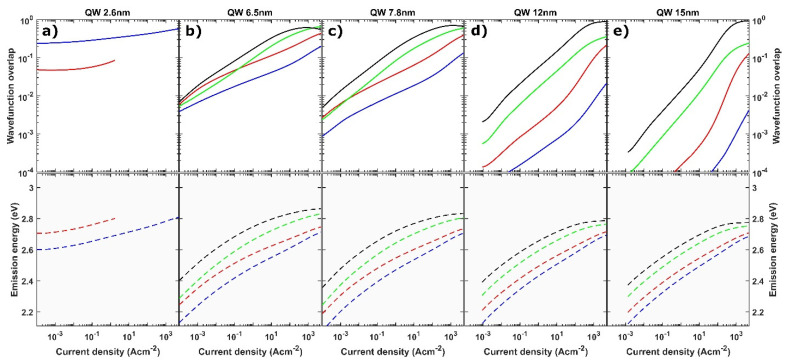
Calculated dependence of wavefunction overlap and peak wavelength emission of various states inside the QW on current density. The QW thickness varies from (**a**) 2.6 nm, (**b**) 6.5 nm, (**c**) 7.8 nm, (**d**) 12 nm and (**e**) 15 nm.

The wavefunction overlaps were calculated for 2.6, 6.5, 7.8, 12 and 15 nm wide QW, and their dependence on current density is shown in Figure 6. The thin, 2.6 nm QW operates only on the transition between the ground states <e1h1>, even though there is also the <e1h2> transition. That is because the h2 excited state is not occupied in the low current regime, and in the high current regime, it is not confined inside the QW (Figure 4a). In the low current regime, the <e1h1> wavefunction overlap is equal to 0.29, whereas at higher currents, in which the polarization field is partially screened, the <e1h1> wavefunction overlap increases up to 0.50 at j = 1000 A cm^−2^.

In the case of LEDs with intermediate and wide QWs, the wavefunction overlap of transitions, including excited states (<e1h2>, <e2h1> and <e2h2>), are higher than the <e1h1> transition. There are two reasons for this situation. The first one is observed in the low carrier density regime, where the QW has a triangular shape and the higher energy states is distrusted along a higher width. This changes dramatically in the high carrier density regime, where the built-in field is strongly screened. The QWs at high injection are no longer triangular, but have a rather complex shape with a high electric field at the edges of the QW and almost no field in the middle of the QW. Such a shape strongly changes the distribution of the excited states, but not the distribution of the ground states. This is especially well observed in the case of the 15 nm QW at high injection (see Figure 4c) in which the e2 state is distributed along the whole width of the QW, whereas the e1 state is localized in the vicinity of the interface. The wavefunction overlaps of the <e1h1> and <e2h2> transitions are equal to 0.001 and 0.81 at j = 1000 A cm^−2^, respectively. The <e2h2> transition is, therefore, a very efficient one. Surprisingly, it has a higher overlap than the <e1h1> transition in the 2.6 nm wide QW.

**Figure 7:**
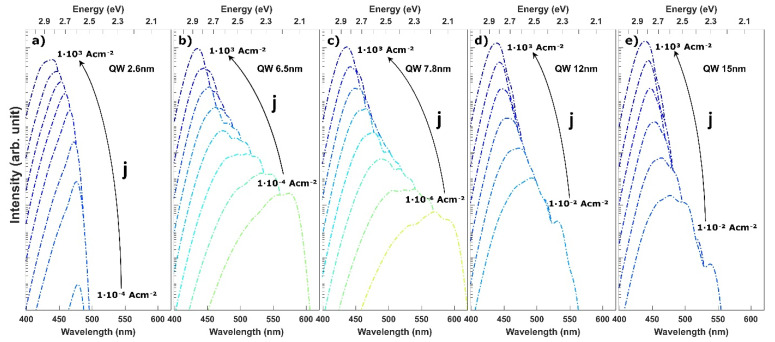
Calculated spectra for InGaN LEDs utilizing a QW with thickness of (**a**) 2.6 nm, (**b**) 6.5 nm, (**c**) 7.8 nm, (**d**) 12 nm and (**e**) 15 nm.

In Figure 7, the calculated emission spectra of LEDs with QW thickness ranging from thin 2.6 nm to very wide 15 nm are presented for various current densities. For the thin QW (Figure 7a) the emission spectra are blue shifted with the current density due to the screening of the polarization charges and the band filling effect. For the intermediate QW thickness of 6.5 nm and 7.8 nm, multiple peak emission spectra can be seen in Figure 7b,c, with the peak wavelength reaching the green-yellow regime. Depending on the current density, different transitions have the highest intensity as can be seen in the case of the 7.8 nm wide QW. The emission evolves to a single peak emission from the <e2h2> transition for high drive currents densities, when all lower states are occupied and the internal electric field is strongly screened.

Based on the insight given by the simulations, we now interpret the experimental results. In the case of the LED with thin QW, the emission did not shift initially because the wavefunction overlap of the <e1h1> transition was large enough for carriers to recombine. Only after the current density was increased to j = 4.4 A cm^−2^, screening of the polarization field began. On the other hand, in the case of intermediate QWs, the <e1h1> wavefunction overlap was small, which led to a fast increase in the carrier density as shown in Figure 5. This resulted in a fast screening of the polarization field and an observed blue shift from the lowest current densities. Furthermore, based on the calculated transition energies, we attribute the second peak, which appears in the spectra, to the <e2h1> transition, whereas the third peak is assigned to the <e2h2> transition. In the case of wide QWs, the spatial separation between the ground states is so extensive that no recombination through the <e1h1> transition occurs. Instead, carriers accumulate at the edges of the QW. Some carriers escape the QW either by thermionic emission or via recombination on defects. Radiative recombination is observed only after almost complete screening of the polarization field, which causes the emergence of transitions through excited states with high wavefunction overlap. This leads to an increase in the current density at which light emission starts. We attribute the emission to exited states, in particular to the <e2h2> transition, as it has the highest wavefunction overlap.

It has to be pointed out that the differences in operating current between the predicted and measured EL spectra are noticeable. For the 2.6 nm QW, we observe the blue shift in the lower currents in the simulation than in experiment. Similarly, for the intermediate QWs, the ground and mixed transitions are dominant up to j = 1 A cm^−2^ in experiments and only up to j = 0.01 A cm^−2^ in simulations. This difference can be attributed to the usage of default ABC parameters in calculations. The values of the ABC parameters strongly influence the recombination rate and thus the carrier density at a given current density. Nevertheless, the main conclusions from the calculation and experiment remain the same, and the separate maxima in EL spectra can be distinguished and attributed to the corresponding transitions. In the case of very wide QW, simulations also predict multiple peaks in low current densities and shift of the main peak to the higher energies, but in the experiment, there is only one wavelength-stable peak on EL data. Experimentally observed wavelength stability of spectra could be explained by the fully screened electric field prior to excitation. However, the work of Brecha et al. suggests that the internal field is not initially screened [32]. The explanation could be that in a low current regime, the device operates on transitions between higher states (<e3h2>, etc.), which have high overlaps in low current. Then, when the current increases the index of states, through which the transition occurs decreases. As a result, no significant change of the emission wavelength in observed. At a high current, the QW operates on the <e2h2> transition. This behavior is additionally supported by simulation, which predicts that in a low-current regime, the <e2h2> transition should give much longer emission wavelength (see Figure 6e) than that observed in the experiment.

## 4. Conclusions

In this paper, we studied the emission properties of InGaN LEDs with various QW thicknesses. We found that, based on the QW width, three categories can be distinguished: thin, intermediate, and wide QWs. In the case of LEDs with thin QWs, we observed a blue shift of the emission spectra only in a high-current regime. In the case of intermediate QWs, there are two distinctive features. The emission starts in the long wavelength regime and a rapid blue shift is observed, even for the lowest current. Furthermore, as the current is increased, additional peaks in the emission spectrum appear. In the case of the wide QW, we observed yet a different behavior: a single peak is observed, with a stable wavelength in the whole current range.

To explain the origin of differences in the behavior of the LEDs with different QW thicknesses, we simulated operation of the devices, using the self-consistent Poisson–Schrodinger solver. We showed that the differences between the LEDs originate from the built-in polarization and different carrier densities in the QW. In the case of the thin QW, the carrier density is initially low and increases with the current density, resulting in screening of the built-in field and blue shift of the emission spectrum. In the case of the intermediate QW, due to an increased separation of the electron and hole wavefunctions, the carrier recombination is reduced and the carrier density is increased more rapidly than for the thin QWs. This results in a faster screening of the built-in polarization and blue shift. Furthermore, due to the high carrier density, additional transitions through excited states were found. On the other hand, the carrier density in wide QWs is very high, even for the lowest currents. This is a consequence of a near-zero wavefunction overlap between the ground states, which promotes an increase in the carrier density. The built-in electric field is almost entirely screened, and the emission occurs through the excited states. We found that in the case of the wide QW, the transition involving ground states is almost forbidden, while the wavefunction overlap between the excited states is especially high.

These results increase the understanding of the nature of carrier recombination in InGaN-based LEDs and provides recommendations for designing active region with properties suitable for various applications, such as wavelength-stable emitters.

## Data Availability

The data presented in this study are available on request from the corresponding author.
